# Experiences of a National Web-Based Heart Age Calculator for Cardiovascular Disease Prevention: User Characteristics, Heart Age Results, and Behavior Change Survey

**DOI:** 10.2196/19028

**Published:** 2020-08-07

**Authors:** Carissa Bonner, Natalie Raffoul, Tanya Battaglia, Julie Anne Mitchell, Carys Batcup, Bill Stavreski

**Affiliations:** 1 Sydney School of Public Health Faculty of Medicine and Health University of Sydney Sydney Australia; 2 National Heart Foundation of Australia Sydney Australia; 3 National Heart Foundation of Australia Melbourne Australia

**Keywords:** heart age, risk communication, cardiovascular disease prevention, eHealth, behavior change

## Abstract

**Background:**

Heart age calculators are used worldwide to engage the public in cardiovascular disease (CVD) prevention. Experimental studies with small samples have found mixed effects of these tools, and previous reports of population samples that used web-based heart age tools have not evaluated psychological and behavioral outcomes.

**Objective:**

This study aims to report on national users of the Australian heart age calculator and the follow-up of a sample of users.

**Methods:**

The heart age calculator was launched in 2019 by the National Heart Foundation of Australia. Heart age results were calculated for all users and recorded for those who signed up for a heart age report and an email follow-up over 10 weeks, after which a survey was conducted. CVD risk factors, heart age results, and psychological and behavioral questions were analyzed using descriptive statistics and chi-square tests. Open responses were thematically coded.

**Results:**

There were 361,044 anonymous users over 5 months, of which 30,279 signed up to receive a heart age report and 1303 completed the survey. There were more women (19,840/30,279, 65.52%), with an average age of 55.67 (SD 11.43) years, and most users knew blood pressure levels (20,279/30,279, 66.97%) but not cholesterol levels (12,267/30,279, 40.51%). The average heart age result was 4.61 (SD 4.71) years older than the current age, including (23,840/30,279, 78.73%) with an older heart age. For the survey, most users recalled their heart age category (892/1303, 68.46%), and many reported lifestyle improvements (diet 821/1303, 63.01% and physical activity 809/1303, 62.09%). People with an older heart age result were more likely to report a doctor visit (538/1055, 51.00%). Participants indicated strong emotional responses to heart age, both positive and negative.

**Conclusions:**

Most Australian users received an older heart age as per international and UK heart age tools. Heart age reports with follow-up over 10 weeks prompted strong emotional responses, high recall rates, and self-reported lifestyle changes and clinical checks for more than half of the survey respondents. These findings are based on a more engaged user sample than previous research, who were more likely to know blood pressure and cholesterol values. Further research is needed to determine which aspects are most effective in initiating and maintaining lifestyle changes. The results confirm high public interest in heart age tools, but additional support is needed to help users understand the results and take appropriate action.

## Introduction

Heart age calculators are increasingly popular worldwide as a way to engage the public in cardiovascular disease (CVD) risk assessment [1]. Methods vary but heart age is generally calculated by comparing a person’s absolute risk of a heart attack or stroke in the next 5-10 years with a person with *ideal* risk factor levels, such as a nonsmoker with 120 mm Hg systolic blood pressure. If any risk factor is higher than ideal (eg, 140 mm Hg systolic blood pressure), then the result is an older heart age [2]. Some calculators also allow younger heart age if risk factors are below the ideal threshold (eg, 110 mm Hg systolic blood pressure).

Heart age calculators are often used as motivational tools to raise personal awareness about CVD risk factors and prompt follow-up action. Millions of people have used web-based heart age calculators. An international Unilever campaign engaged 2.7 million users from 13 countries in 2009 to 2011 using a Framingham model–based heart age calculator [3], and a more recent UK version based on QRISK reached 1.4 million hits with almost 600,000 complete users over 5 months [4]. In New Zealand, a 5-year Framingham version was used to promote clinical guidelines [2], and health organizations in the United States and China have released population estimates of heart age [5,6]. Australia launched a heart age calculator as part of a national consumer awareness campaign in February 2019, which engaged approximately 1.6 million users over 16 months.

With an increasing number of heart age calculators becoming available on the web, it is important to note that the same person can get a very different heart age result depending on which calculator is used [1]. This depends on the underlying absolute risk model (eg, Framingham vs QRISK), the ideal thresholds set for risk factors (eg, systolic blood pressure 120 mm Hg), and restrictions in the way that absolute risk is converted to heart age (eg, allowing younger heart age or not). It is important to note that an older heart age is not the same as high absolute risk where clinical guidelines would recommend medication; it is an alternative risk communication format that indicates at least one elevated risk factor compared with the ideal levels set. It is therefore possible to have a low absolute risk but an older heart age, for example, a young woman with cholesterol levels above the ideal level.

With so much variability in the way these calculators are set up, heart age is not recommended as a clinical assessment tool for medication decisions [1]. However, they may engage users to consider the personal relevance of risk factors and lifestyle changes or to seek a more accurate risk assessment with their doctor [7]. Particularly in younger adults, low absolute risk may conceal a high relative risk of developing CVD, and heart age calculators can be useful in communicating the long-term consequences of an individual’s lifestyle and associated risk factors [8]. Some trials have found that using a heart age tool improves risk factor control compared with standard care [9], and direct comparisons of heart age with absolute risk in the same interactive format have found greater emotional responses to heart age, but this may not necessarily translate into behavior change [10-12]. Therefore, additional support is needed to help users understand the results and take appropriate action based on heart age calculators.

Existing research on the effect of heart age calculators is divided into small experimental samples that were randomized (which show mixed results overall) [13] and large population samples where users have been simply described. This study aimed to draw these 2 areas together by reporting on the users of a new Australian heart age calculator, followed by lifestyle change outcomes in a smaller sample of users who signed up to receive a report and further support by email. Previous reports describing the general population’s use of heart age calculators have not evaluated behavioral outcomes and include repeated or less serious uses of the tool (eg, just testing the tool or trying it out for someone else).

## Methods

### Materials

After conducting an environmental scan of international heart age calculators, the National Heart Foundation of Australia (NHFA) created the Australian version based on Framingham model algorithms. The calculator was developed with funding from an unrestricted and unconditional grant from Amgen, who did not contribute in any way to the development. Some adjustments were made in line with Australian guidelines (eg, ideal levels set at 120 mm Hg for systolic blood pressure and <4 mmol/L for total cholesterol), which resulted in some changes to the weightings for some gender or age groups in the published model. These were tested and discussed with a committee including general practitioners (GPs) and cardiologists to ensure that the calculator would not potentially lead to treatment based on single risk factors. A pop-up message prompted users to see a doctor if the blood pressure or cholesterol level was plausible but considered high risk in accordance with Australian guidelines, for example, “Total cholesterol above 7.5 mmol/L puts you at high risk of having a heart attack or stroke. Please see your doctor as soon as possible about your cholesterol.” Implausible values prompted a different message about the range required, for example, “Please enter a number between 2 and 10.5.” Heart age was calculated once a plausible value was entered. No adjustments were made to account for higher risk populations (eg, Aboriginal and Torres Strait Islander Peoples) because of a lack of clear evidence. The minimum reported heart age was set at <35 years and the maximum at ≥85 years. If blood pressure or cholesterol levels were not known, a population average was used based on the relevant 5-year age group in the National Health Survey data from 2011 to 2012 [14].

**Figure 1:**
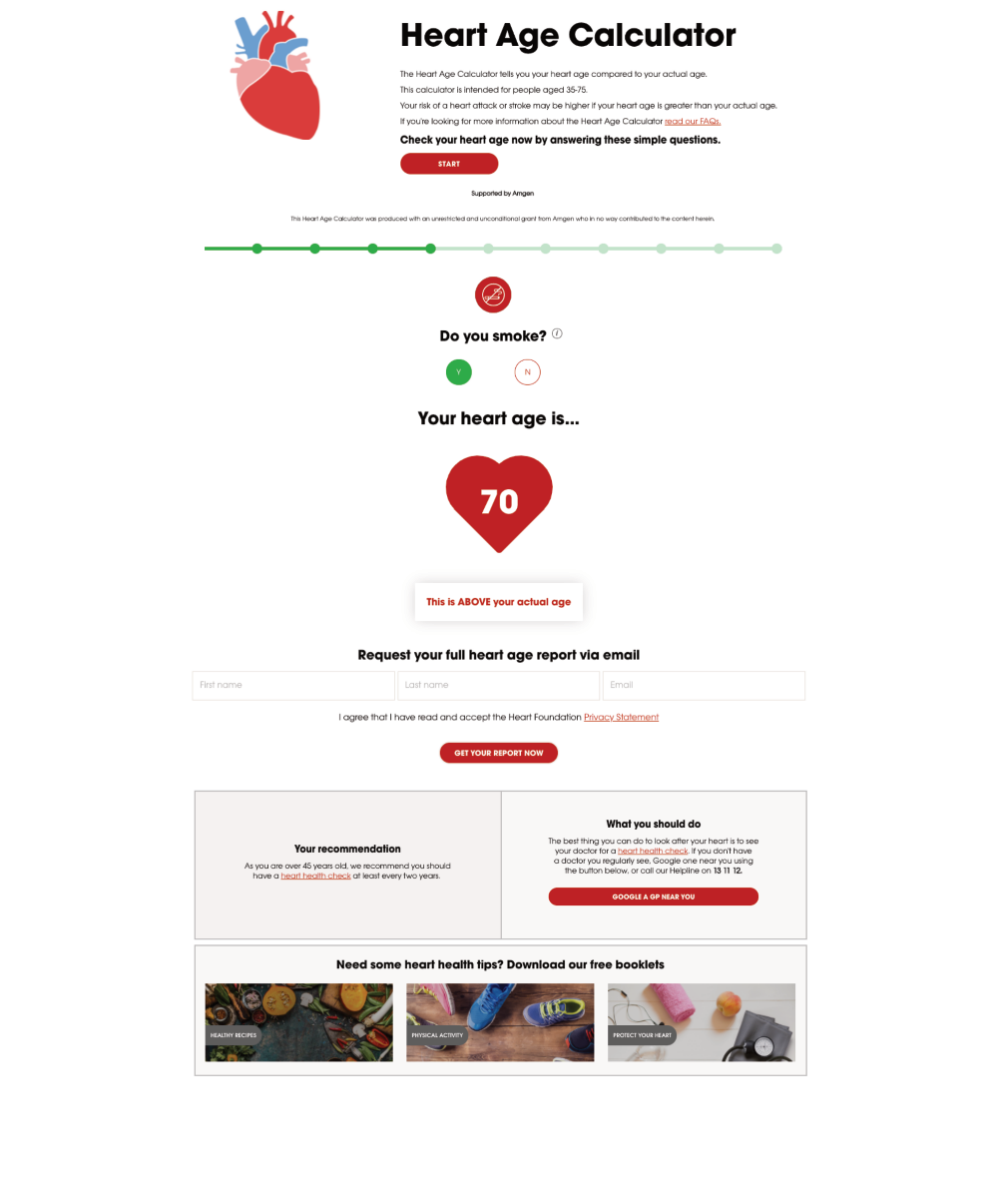
Example screenshots from heart age calculator (eg, 54 year old male smoker, family history, diabetes and average blood pressure/cholesterol).

**Figure 2:**
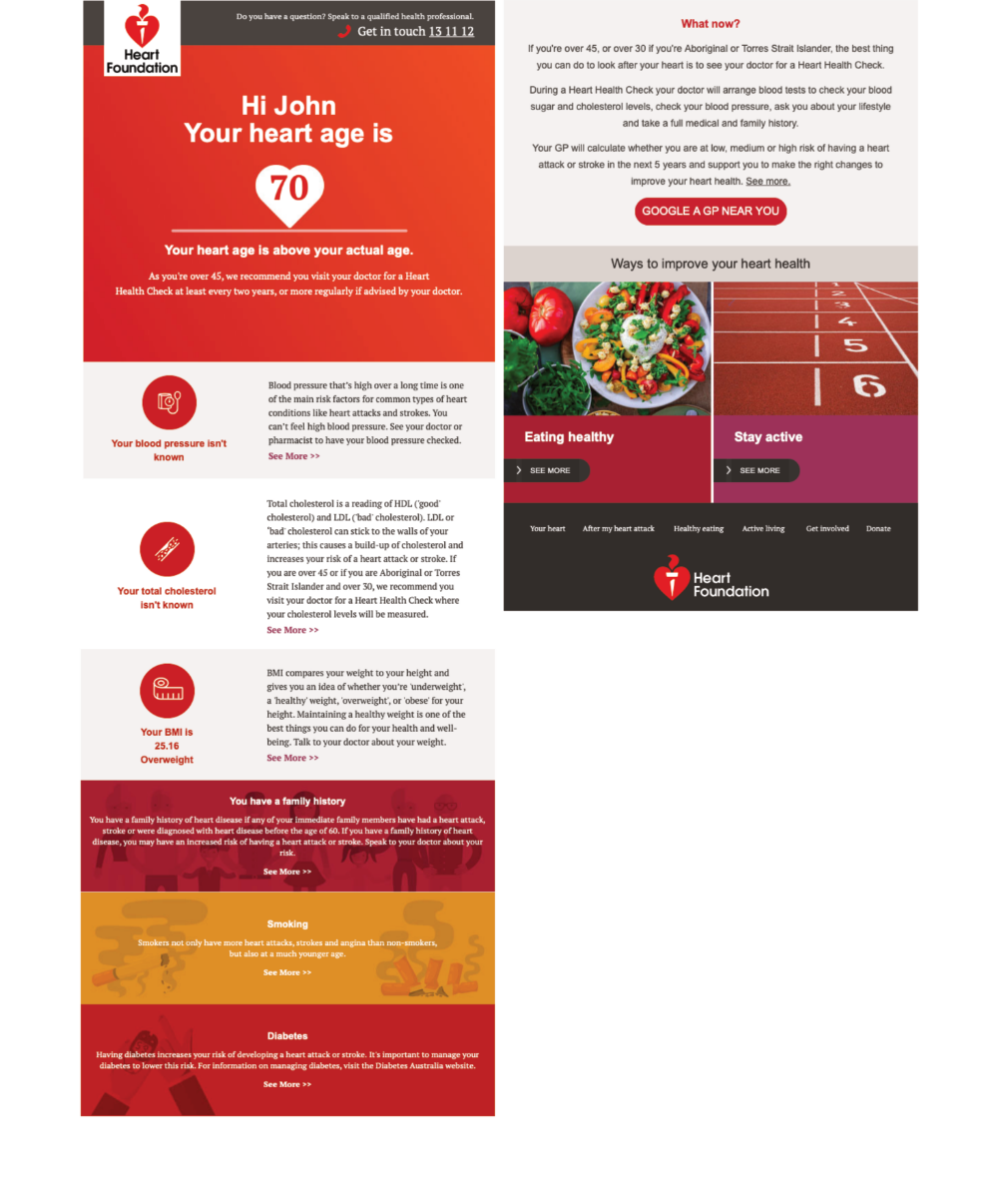
Example heart age calculator report (eg, 54 year old male smoker, family history, diabetes, and average blood pressure/cholesterol).

The resulting web-based heart age calculator was intended for people aged 35 to 75 years without existing CVD. The following information was collected: age, sex, family history of premature heart disease, smoking status, height, weight, diabetes status, blood pressure, cholesterol, and whether or not users were taking medication for high blood pressure. Users who did not know their blood pressure or cholesterol were informed that a population average would be used. The result was presented as the user’s heart age and whether it was younger, the same as, or older than their current age. Users were encouraged to provide their email address to obtain a more detailed report and those in the target age group were recommended to see a doctor for a heart health check for absolute risk assessment. The tool is available on the NHFA website [15]. Figure 1 shows example screenshots, and Figure 2 shows example reports.

### Procedure

**Figure 3:**
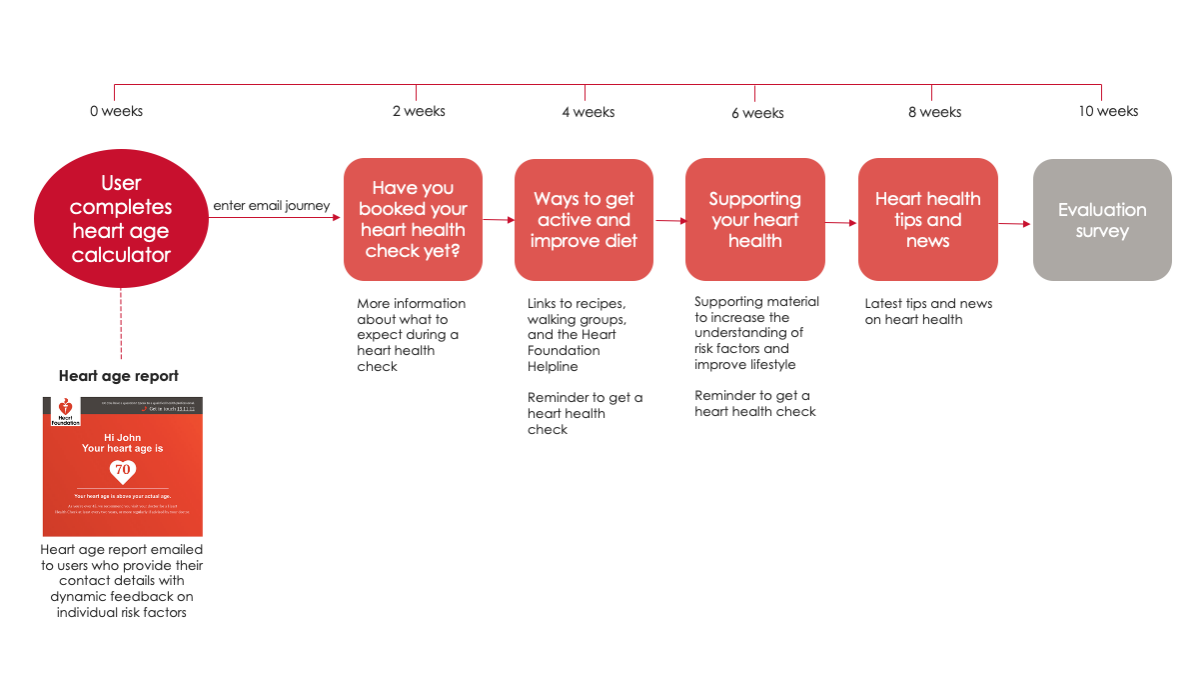
Heart age calculator email journey flowchart.

The Australian heart age calculator was launched in February 2019 as part of an NHFA consumer awareness campaign involving mass marketing and media interventions. This *Serial Killer* campaign aimed to boost public awareness around heart disease as Australia’s leading cause of death and sought to increase the personal relevance of the condition to all Australian adults. Other campaign objectives included advocating for a range of Heart Foundation federal election requests, including Medicare-funded heart health checks. The web-based heart age calculator URL was included as a *call to action* for this campaign. After completing the heart age calculator, users were prompted to sign up to receive a detailed report via email, further explaining their heart age results (Figure 2). Users were then automatically signed up to a 10-week email journey consisting of fortnightly emails prompting eligible patients to see their GP for a heart health check and providing general advice on healthy eating, exercise, and heart health (Figure 3). The email journey included various behavior change techniques [16], namely: credible source, prompts/cues, goal setting, information about health consequences, salience of consequences, instruction on how to perform the behavior, social support, and material incentive (reward for completing a lifestyle challenge via an app). At 10 weeks, users were asked to participate in a follow-up survey to evaluate psychological and behavioral outcomes, including recall, positive and negative emotional responses, information seeking, lifestyle change, and clinical checks. Survey respondents entered a draw to win 1 of 3 gift cards. An open response question was also included to evaluate general reactions to heart age. Multimedia Appendix 1 provides all survey questions.

### Participants

The calculator could be completed by Australians aged 35-75 years without CVD, in accordance with the target group for CVD risk assessment in Australia.

### Analysis

User data were cleaned to remove duplicates based on internet protocol addresses or email addresses, and users who completed the heart age calculator between February 19 and July 31, 2019, were included in the final data set. CVD risk factors (for all anonymous users), heart age results (for those who requested a report by email), and psychological and behavioral questions (for survey respondents) were linked to the original heart age calculator results. Statistical analysis was performed using IBM SPSS Statistics version 26 (IBM Corp) statistical software package (TB). Descriptive statistics are reported with numbers and percentages for the 3 samples, and exploratory comparisons between age, gender, and heart age category groups in the survey sample were performed using chi-square tests, where a value of *P*<.05 was considered statistically significant. Free text responses to the heart age result were coded using a framework analysis approach where themes were first identified from the data using an inductive approach (C Batcup and C Bonner). We then applied a theoretical framework deductively to organize the themes (C Bonner), before all data were coded under the categories of expectation, experience, risk perception, evaluation, and action (C Batcup). The framework was developed in a previous qualitative study using think-aloud methods to understand how participants use and react to heart age calculators [7]. A sample of 10% was double coded, and discrepancies were resolved through discussion (C Batcup and C Bonner). All authors contributed to the interpretation of the results.

### Ethical Approval

An exemption letter was provided by the University of Sydney Human Research Ethics Committee, as the study involved an analysis of existing anonymized data, originally obtained by the NHFA for internal evaluation purposes.

## Results

### CVD Risk Factors for Anonymous Users, Those Who Requested a Report, and Survey Respondents

**Figure 4:**
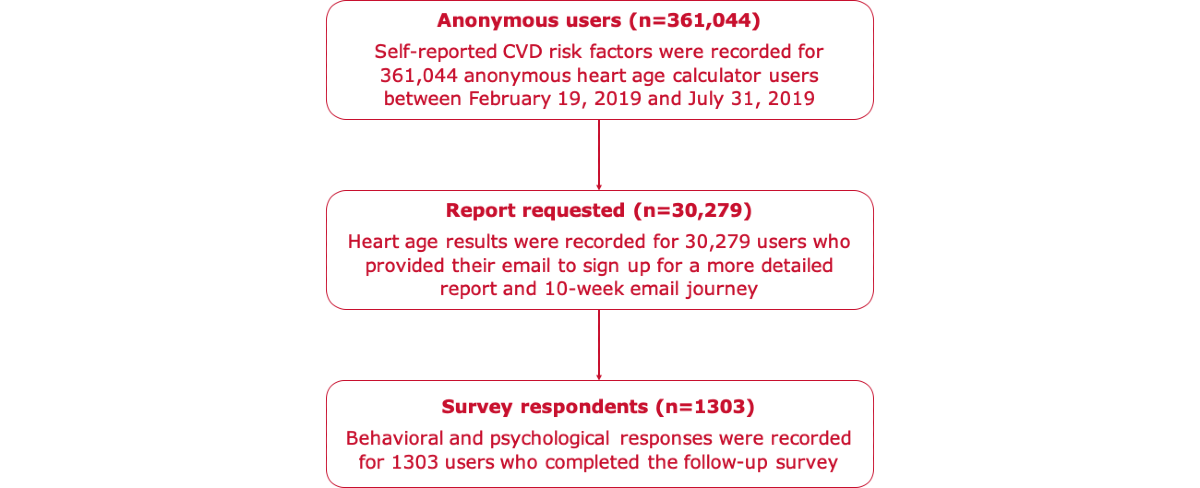
Sample flowchart.

Overall, data were obtained from 361,044 anonymous heart age calculator users (CVD risk factors only), 30,279 users who provided email addresses to request a report (heart age results) and 1303 survey respondents (psychological and behavioral questions). Figure 4 shows a sample flowchart, and Table 1 provides a summary of risk factors for the 3 samples. The anonymous user sample was younger (mean 49.37, SD 11.79 years) with a higher proportion of smokers (35,503/361,044, 9.83%), and fewer knew their blood pressure level (178,281/361,044, 49.38%) and cholesterol level (59,013/361,044, 16.35%), were on blood pressure–lowering medication (64,464/361,044, 17.85%), and reported a family history (123,680/361,044, 34.26%). Of those who provided their email to receive the report and follow-up, there were 19,840 (19,840/30,279, 65.52%) women and 10,439 (10,439/30,279, 34.48%) men, with 80.40% (24,348/30,279) in the target age for heart health checks for the general population (45-75 years), and a mean age of 55.67 (SD 11.43) years. In terms of modifiable risk factors, 6.46% (1957/30,279) of users reported smoking, 40.51% (12,267/30,279) knew their cholesterol level, 66.97% (20,279/30,279) knew their blood pressure level, and 26.26% (7950/30,279) were taking blood pressure–lowering medication. Less than half of the users (12,844/30,279, 42.42%) reported a family history of heart disease, and only 7.56% (2290/30,279) of users reported having a diagnosis of diabetes. The survey respondent sample was older (mean age 60.43, SD 10.15 years), with a lower proportion of smokers (39/1303, 2.99%); and more knew their blood pressure level (961/1303, 73.75%), knew their cholesterol level (585/1303, 44.90%), were on blood pressure–lowering medication (413/1303, 31.70%), and reported a family history (587/1303, 45.04%).

**Table 1:** Risk factors by heart age calculator user sample.

CVD^a^ risk factors			Anonymous users (n=361,044)		Report requested (n=30,279)		Survey respondents (n=1303)	
**Gender, n (%)**								
	Female			221,278 (61.29)		19,840 (65.52)		867 (66.54)
	Male			139,766 (38.71)		10,439 (34.48)		436 (33.46)
**Age (years)**								
	Mean (SD)			49.37 (11.79)		55.67 (11.43)		60.43 (10.15)
	**Range, n (%)**							
		35-44		144,430 (40.00)		5931 (19.59)		112 (8.60)
		45-54		88,945 (24.63)		7015 (23.17)		216 (16.58)
		55-64		83,313 (23.08)		9809 (32.40)		469 (35.99)
		65-75		44,356 (12.29)		7524 (24.85)		506 (38.83)
Smoker, n (%)			35,503 (9.83)		1957 (6.46)		39 (2.99)	
Family history of CVD, n (%)			123,680 (34.26)		12,844 (42.42)		587 (45.04)	
Diabetes, n (%)			20,606 (5.71)		2290 (7.56)		89 (6.83)	
Taking BP^b^ medication, n (%)			64,464 (17.85)		7950 (26.26)		413 (31.70)	
Know BP level, n (%)			178,281 (49.38)		20,279 (66.97)		961 (73.75)	
Know cholesterol level, n (%)			59,013 (16.35)		12,267 (40.51)		585 (44.90)	

^a^CVD: cardiovascular disease.

^b^BP: blood pressure.

### Heart Age Results for Users Who Requested a Report

Overall, heart age was on average 4.61 years older than current age, including 78.73% (23,840/30,279) with older heart age and 13.75% (4163/30,279) with younger heart age. Heart age results were significantly different by age group (χ^2^_6_=1601.1; *P*<.001) and gender (χ^2^_2_=445.2; *P*<.001). Those aged 44-54 years were the most likely group to receive a younger heart age for both women (1380/4640, 29.74%) and men (266/2375, 11.20%), whereas those aged 64-75 years were the most likely group to receive an older heart age result for women (4442/4812, 92.31%) and men (2440/2712, 89.97%). Women were almost twice as likely to receive a younger heart age result than men overall (3242/19,840, 16.34% vs 921/10,439, 8.82%).

### Psychological and Behavioral Outcomes for the Survey Respondent Sample

Compared with the total sample that requested a report, survey respondents had a slightly higher proportion of people with an older heart age result (1055/1303, 80.97% vs 23,840/30,279, 78.73%) and a slightly lower proportion of people with a younger heart age result (155/1303, 11.90% vs 4163/30,279, 13.75%), but the rates were similar.

Table 2 summarizes the psychological and behavioral outcomes for the survey respondent sample. Of those who completed the survey 10 weeks after their initial result, most (892/1303, 68.46%) were able to correctly recall their heart age category as being younger, equal to, or older than their current age. This was similar for younger (104/155, 67.09%) and older (735/1055, 69.67%) heart age results, but significantly lower for equal heart age results (53/93, 56.99%; χ^2^_2_=6.5; *P*=.04). More than one-fourth of users reported feeling a strong positive emotional response (507/1303, 38.91% very motivated and 324/1303, 24.87% very optimistic), and a lower proportion reported strong negative emotions (167/1303, 12.82% very anxious and 160/1303, 12.28% very worried). Compared with those with a younger/equal heart age, users who received an older heart age report were more likely to feel very anxious (159/1055, 15.07% vs 8/248, 3.2% reporting a great deal or a lot; χ^2^_1_=25.2; *P*<.001) or worried (151/1055, 14.31% vs 9/248, 3.63%; χ^2^_1_=21.3; *P*<.001). They were less likely to feel optimistic about their result (229/1055, 21.71% vs 95/248, 38.3%; χ^2^_1_=29.6; *P*<.001), but motivation levels were similar (406/1055, 38.48% vs 101/248, 40.7%; χ^2^_1_=0.4; *P*=.52).

In terms of lifestyle behavior, more than half of the survey respondents reported improvements in their diet (821/1303, 63.01%) and physical activity (809/1303, 62.09%), with just under half reporting weight loss (643/1303, 49.35%). Almost one-third of users reported reducing stress (412/1303, 31.62%) and alcohol intake (406/1303, 31.16%). Of those who smoked, 48% (19/39) reported reductions. Some lifestyle change behaviors were reported at higher rates for those with older compared with younger/equal heart age, including diet (680/1055, 64.45% vs 141/248, 56.8%; χ^2^_1_=5.0; *P*=.03) and weight loss (537/1055, 50.90% vs 106/248, 42.7%; χ^2^_1_=5.4; *P*=.02).

For outcomes relating to clinical risk assessment, almost half of the users had already seen their GP (621/1303, 47.66%), and one-fourth reported receiving a heart health check (362/1303, 27.78%) in the 10 weeks since receiving their heart age report. Higher proportions had obtained specific clinical tests, with three-fourths of the users checking blood pressure level and more than half obtaining blood tests for cholesterol (737/1303, 56.56%) and diabetes or sugar levels (697/1303, 53.49%). People with an older heart age result were more likely to have visited their doctor (538/1055, 51.00% vs 83/248, 33.4%; χ^2^_1_=24.7; *P*<.001) or had a heart health check (314/1055, 29.76% vs 48/248, 19.3%; χ^2^_1_=10.8; *P*<.001), compared with those with a younger or equal heart age.

**Table 2:** Heart age calculator user outcomes after 10 weeks for survey respondents.

Outcomes		All survey respondents (n=1303), n (%)		Older heart age (n=1055), n (%)		Younger or equal heart age (n=248), n (%)	
**Psychological**							
	Recall of correct heart age category		892 (68.4)		735 (69.6)		157 (63.3)
	Very motivated (a great deal/a lot)		507 (38.9)		406 (38.4)		101 (40.7)
	Very optimistic (a great deal/a lot)		324 (24.8)		229 (21.7)		95 (38.3)
	Very anxious (a great deal/a lot)		167 (12.8)		159 (15.0)		8 (3.2)
	Very worried (a great deal/a lot)		160 (12.2)		151 (14.3)		9 (3.6)
	Spoke to family about familial history		555 (42.5)		466 (44.1)		89 (35.8)
	Found out more information		787 (60.4)		669 (63.4)		118 (47.5)
	Told others about the calculator		492 (37.7)		397 (37.6)		95 (38.3)
**Lifestyle change**							
	Increased physical activity		809 (62.0)		668 (63.3)		141 (56.8)
	Lost weight		643 (49.3)		537 (50.9)		106 (42.7)
	Improved diet		821 (63.0)		680 (64.4)		141 (56.8)
	Reduced or quit smoking		19 (48.7)		19 (48.7)		0 (0.0)
	Reduced stress		412 (31.6)		333 (31.56)		79 (31.85)
	Limited alcohol intake		406 (31.1)		332 (31.4)		74 (29.8)
**Clinical risk assessment**							
	Saw general practitioner		621 (47.6)		538 (51.0)		83 (33.4)
	Had a heart health check up		362 (27.7)		314 (29.7)		48 (19.3)
	Had a blood pressure check		976 (74.9)		809 (76.6)		167 (67.3)
	Had a blood test for cholesterol		737 (56.5)		619 (58.6)		118 (47.5)
	Had a test for diabetes or sugar levels		697 (53.4)		587 (55.6)		110 (44.3)

### Qualitative Responses to Heart Age for the Survey Respondent Sample

The 1077 open response comments were coded and organized into 5 themes from a previous qualitative study on the process of heart age calculator use [7]: (1) participants’ expectations of what the result would show, (2) their experience of seeing the result, (3) what they understood about their risk based on the results, (4) their evaluation of the result as a credible source of information, and (5) actions they were prompted to take. These themes and their subthemes are shown in Table 3, along with sample quotes. Those with older heart age tended to show more concern about the result and considered it as an indication of ill health or a need for change, although some thought it was not a problem or disregarded the result. Those with younger or equal heart age described feeling happier about their result and considered their health to be good whereas some wanted it to be lower. Many participants also provided reasons for why they received their result, citing a variety of factors such as fitness levels, genetics, and poor overall health. Common actions prompted by the heart age calculator included arranging a GP consultation, changing diet, or increasing physical activity.

**Table 3:** Themes identified in open responses to heart age results.

Themes and subthemes		Example quotes
**Expectations**		
	Perception of lifestyle	“I'm a bit unsure why as I exercise regularly, don't smoke only drink occasionally, within normal weight range”
	Information from doctor	“I had only just had an appointment with my cardiologist and he said my heart is very good”
**Experience**		
	Happy or fine with result	“I'm on the right track”
	Surprise at result	“Surprised and puzzled as to true meaning”
	Concerned or disappointed	“I was quite shocked and worried”
	Defensive at result	“How is it possible as I had the best possible score therefore everybody must be above”
	Focus on age or being old	“It still feels old!”
	No impression	“I didn't think that it had any relevance to me”
**Risk perception**		
	Indicates good health	“I assumed it meant that my heart was probably in good condition for my age”
	Indicates health issues	“I have a higher than average chance of having a heart attack or a stroke”
	Unsure of meaning	“Don't really know”
	Inconsistent with heart age category	“That I am healthier than average (older heart age result).” “I am unhealthy (younger heart age result)”
	Interpretation reflects heart age category	“Heart is older than my age (older heart age result).” “My heart is in better health than it's [SIC] actual age (younger heart age result)”
	Current or heart age discrepancy	“79 was extremely scary for a 67 year old (older heart age result).” “It was only one year younger, so it was good, but not great (younger heart age result)”
**Evaluation**		
	Incorrect or mistrust result	“The assessment tool was too simplistic to be reliable”
	Expected result	“I was aware that this would probably be the case”
	Risk factors too limited	“I was annoyed as the questions were quite limited and did not take account lifestyle and medications”
	Family history or genetics	“I thought it was elevated because of my family history because I otherwise take good care of my health”
	Explain result	“I was not eating properly and exercising enough”
**Action**		
	No motivation to change	“I'm on track with my general health”
	Need to change	“I thought it meant I had to do some work to get it back to my right age or lower”
	See a doctor	“That I needed to see a doctor”
	Reflection on life	“An aged heart that hasn't been well taken care of. A wake up call to nuture [*SIC*] it and the rest of me”

## Discussion

### Principal Findings

This paper is the first report of a national Australian sample of heart age calculator users. It contributes to the broader heart age outcomes literature with a larger sample of population users, who requested a report with follow-up to support behavior change over a 10-week period. In line with other tools used internationally and in the United Kingdom [3,4], the majority of users who requested a heart age report received an older heart age, but many people did not know their cholesterol or blood pressure levels; therefore, the risk assessment was often based on the population average. More than half of the survey respondents reported lifestyle changes after using the heart age calculator, and many reported seeking further information, including clinical checks to receive a more reliable risk assessment, particularly if they received an older heart age. Interestingly, changes were also reported by those who received younger and equal heart age results. This aligns with prior qualitative research, showing that the process of using heart age calculators can prompt the consideration of lifestyle changes regardless of the actual result [7]. This finding could be because of the process of receiving a heart age result regardless of its value or alternatively the survey sample could have been more motivated in general, that is, they were interested in lifestyle changes before the heart age result. However, a previous randomized study found psychological differences between the heart age and control groups that were similar for younger and older heart age results, suggesting that there is something about receiving this risk format that does prompt different reactions regardless of the result itself [10].

The Australian heart age calculator website has been accessed by a large number of people, with 1.3 million users engaged in the first year (internal figures from the NHFA). This paper shows that older people, those more likely to know their risk factors and/or take medication and nonsmokers, were more likely to engage further in health promotion activities via a digital follow-up report. This points to the need for additional strategies to engage people with unknown risk factors and some high-risk groups. Alternative biological age concepts such as *lung age* may be more effective for engaging specific groups, such as younger smokers [17]. The high prevalence of web-based health risk calculators (eg, CVD, diabetes, and cancer [18-20]) shows that this is a popular marketing strategy or call to action, which may be effective if targeted to the right audience and backed up with behavior change support programs. Systematic reviews show that risk communication can increase intentions to change health-related behavior, and effects on behavior can be enhanced by addressing several aspects of risk perception and repeated communication [21,22]. However, additional behavior change techniques may be needed to bridge the intention-behavior gap and maintain changes over time, such as action plans that incorporate implementation intentions [23,24].

As found in previous experiments comparing heart age to absolute risk [10,11], this sample reported high recall of the heart age result category and strong emotional responses to concepts such as worry about older heart age or optimism. Those who received equal heart age were less likely to remember this, suggesting that positive responses to young heart age or negative responses to older heart age may reinforce the result and aid recall. Previous research has also raised the issue of credibility [7,10], which was reflected in the thematic analysis of open responses. Users had many questions about the role of additional risk factors, conflicting information from health professionals, and the reliability of the web-based assessment, particularly when the result was unexpected. This led some participants to question the usefulness of the heart age calculator but prompted others to seek clinical assessments or lifestyle changes. For heart age calculator developers, it may be important to explain how and why different risk factors are used for those who want more explanation and to clearly state the need to see a doctor for a more accurate risk assessment.

The launch of the Australian heart age calculator was part of a broader campaign to address barriers to absolute CVD risk assessment, including lobbying for federal government funding of clinical heart health checks. More than half of the survey respondents reported having seen their GP in the 10 weeks since finding out their heart age, and one-fourth of users reported receiving a heart health check. Most users were eligible for a full CVD risk assessment with their GP in line with clinical guidelines targeting those aged 45-74 years. The barriers to engage otherwise healthy adults in preventive health checks are complex, covering all 3 broad determinants of behavior change: capability (eg, lack of knowledge and awareness), opportunity (eg, time and access constraints), and motivation (eg, aversion to preventive medicine) [25,26]. Heart age calculators may be particularly useful for addressing awareness issues and motivating people to see their doctor for a more accurate clinical assessment, but this needs to be supported by the broader health system to address opportunity barriers. In Australia, a combination of strategies has led to more than 100,000 Australians receiving a heart health check from their GP under the Medicare Benefits Scheme in the 12 months since this heart age calculator was launched [27].

Further research is needed to determine whether the behavioral outcomes of heart age calculators can be improved by linking it to additional behavior change strategies known to improve lifestyle change (eg, action planning) [16] and whether absolute risk formats used in clinical practice can be equally engaging [28]. There is very little research comparing different labels for the general concept of *biological age*, but one study has found differences in the way that young people interpret *heart age* compared with *fitness age* even when the same numerical age result is used [29]. Different target populations may respond best to different labels, and it is important to consider potential harms from misunderstandings as well as the potential for positive behavior changes. Further investigation is also needed to address the information needs of people with lower health literacy, who have fewer skills required to access, understand, and act on health information [30]. Different interactive tools may be needed for different patient populations to enable informed consent about CVD management options, such as patient decision aids with actionable values clarification exercises to help people weigh lifestyle approaches compared with medication recommended by a doctor [31].

### Strengths and Limitations

The main strength of this study is the analysis of a more engaged sample than other national/international heart age user reports (excluding repeat and nonserious users), but the survey respondents are likely biased in terms of motivation and have different characteristics to the broader samples in this study. As there was no randomization, we could not determine causation or efficacy of heart age over other risk communication methods or the length of follow-up required for sustained lifestyle change. The descriptive data available could not be used to determine whether the heart age result itself caused behavior change or whether it simply promoted engagement with further behavior change strategies.

In conclusion, the results confirm high public interest in heart age tools as a way to engage people in the target age for CVD risk assessment and prevention activities, with the potential to prompt clinical risk assessments and lifestyle changes for many users. Supporting the initial heart age result with more detailed reports to explain the results and evidence-based behavior change techniques may improve the effectiveness of these tools.

## Appendix

Multimedia Appendix 1 Follow up survey items.

## Abbreviations

BP: blood pressure
CVD: cardiovascular disease
GP: general practitioner
NHFA: National Heart Foundation of Australia
