# Supplementary material for: Experiences of a National Web-Based Heart Age Calculator for Cardiovascular Disease Prevention: User Characteristics, Heart Age Results, and Behavior Change Survey
Source: J Med Internet Res. 2020 Aug 7;22(8):e19028. doi: 10.2196/19028 (PMC7442940; doi:10.2196/19028)
Supplement: Multimedia Appendix 1 [file jmir_v22i8e19028_app1.pdf]

Heart Age Calculator – follow-up survey

Q1.	Was your heart age above, equal or below your actual age?	Above ....1<br>Equal..2<br>Below..3<br>Can't remember 98

Q2.	When first finding out your heart age was above, equal or below your actual age, what did you think it meant?	Please specify<br>Unsure.. 98

Q3. To what extent did your heart age results make you feel…?						
	Not at all	A little	A moderate amount	A lot	A great deal	Unsure
Motivated	1	2	3	4	5	98
Optimistic	1	2	3	4	5	98
Anxious	1	2	3	4	5	98
Worried	1	2	3	4	5	98

Q4.	After using the Heart Age Calculator, would you say your understanding of your risk of a heart attack or stroke is…?	Much better ....1<br>Somewhat better ....2<br>The same ....3<br>Somewhat worse..4<br>Much worse..5<br>Unsure.. 98

Q5. Since finding out your heart age, have you…?				
	Yes	No, but I am planning on doing this	No, I have not thought about doing this at all	Not applicable / Can't remember
Visited your doctor	1	2	3	98
Had a blood pressure check	1	2	3	98
Had a blood test for cholesterol	1	2	3	98
Had a test for diabetes or high blood sugar levels	1	2	3	98
Had a heart health check up (where your doctor estimated your risk of having a heart attack or stroke in the next 5 years)	1	2	3	98

Q6. Since finding out your heart age, have you…?			
	Yes	No	Not applicable / Can't remember

Found out more, or intend to find out more about risk factors for heart disease	1	2	98
Spoke to family members about the history of heart disease in your family	1	2	98
Told family and friends about the Heart Age Calculator	1	2	98
Contacted the Heart Foundation Helpline	1	2	98

Q7. Since finding out your heart age, what changes, if any, have you made to manage your heart health?

	I'm planning on doing this	I have started doing this	I have not thought about this at all	Not applicable	Unsure
Increasing physical activity	1	2	3	99	98
Losing weight	1	2	3	99	98
Improving your diet (e.g. increasing fruit, vegetable and fibre intake)	1	2	3	99	98
Reducing / quitting smoking	1	2	3	99	98
Reducing stress	1	2	3	99	98
Limiting your alcohol intake	1	2	3	99	98
Having regular checks on blood pressure	1	2	3	99	98
Having regular checks on cholesterol levels	1	2	3	99	98
Taking blood pressure medication	1	2	3	99	98
Taking cholesterol medication	1	2	3	99	98

Q8. Which of the following age groups do you fall into?

Under 35 1
35 to 39...2
40 to 44...3
45 to 49...4
50 to 54...5
55 to 59...6
60 to 64...7
65 to 69...8
70 to 75...9
Over 75 10

Q9. Are you …?

Male 1
Female 2
